# Common variants of *NFE2L2* gene predisposes to acute respiratory distress syndrome in patients with severe sepsis

**DOI:** 10.1186/s13054-015-0981-y

**Published:** 2015-06-16

**Authors:** Marialbert Acosta-Herrera, Maria Pino-Yanes, Jesús Blanco, Juan Carlos Ballesteros, Alfonso Ambrós, Almudena Corrales, Francisco Gandía, Carlés Subirá, David Domínguez, Aurora Baluja, José Manuel Añón, Ramón Adalia, Lina Pérez-Méndez, Carlos Flores, Jesus Villar, for the GRECIA and GEN-SEP networks

**Affiliations:** CIBER de Enfermedades Respiratorias, Instituto de Salud Carlos III, Madrid, Spain; Research Unit, Hospital Universitario Nuestra Señora de Candelaria, Carretera del Rosario 145, 38010 Santa Cruz de Tenerife, Spain; Multidisciplinary Organ Dysfunction Evaluation Research Network, Research Unit, Hospital Universitario Dr. Negrín, Barranco de la Ballena s/n – 4th floor, south wing, 35019 Las Palmas de Gran Canaria, Spain; Intensive Care Unit, Hospital Universitario Río Hortega, Valladolid, Spain; Intensive Care Unit, Hospital Clínico Universitario de Salamanca, Salamanca, Spain; Intensive Care Unit, Hospital General Universitario de Ciudad Real, Ciudad Real, Spain; Intensive Care Unit, Hospital Clínico de Valladolid, Valladolid, Spain; Intensive Care Unit, Fundació ALTHAIA, Manresa, Spain; Department Anesthesia, Hospital Universitario Nuestra Señora de Candelaria, Santa Cruz de Tenerife, Spain; Department Anesthesiology, Hospital Clínico Universitario, Santiago de Compostela, Spain; Intensive Care Unit, Hospital Virgen de La Luz, Cuenca, Spain; Department of Anesthesiology, Hospital Clinic de Barcelona, Barcelona, Spain; Applied Genomics Group, Laboratory of Genetics, Instituto Universitario de Enfermedades Tropicales y Salud Pública de Canarias, Universidad de La Laguna, Tenerife, Spain; Keenan Research Center for Biomedical Science, St. Michael’s Hospital, Toronto, ON Canada

## Abstract

**Introduction:**

The purpose of this study was to investigate whether common variants across the nuclear factor erythroid 2-like 2 (*NFE2L2*) gene contribute to the development of the acute respiratory distress syndrome (ARDS) in patients with severe sepsis. *NFE2L2* is involved in the response to oxidative stress, and it has been shown to be associated with the development of ARDS in trauma patients.

**Methods:**

We performed a case–control study of 321 patients fulfilling international criteria for severe sepsis and ARDS who were admitted to a Spanish network of post-surgical and critical care units, as well as 871 population-based controls. Six tagging single-nucleotide polymorphisms (SNPs) of *NFE2L2* were genotyped, and, after further imputation of additional 34 SNPs, association testing with ARDS susceptibility was conducted using logistic regression analysis.

**Results:**

After multiple testing adjustments, our analysis revealed 10 non-coding SNPs in tight linkage disequilibrium (0.75 ≤ *r*^*2*^ ≤ 1) that were associated with ARDS susceptibility as a single association signal. One of those SNPs (rs672961) was previously associated with trauma-induced ARDS and modified the promoter activity of the *NFE2L2* gene, showing an odds ratio of 1.93 per T allele (95 % confidence interval, 1.17–3.18; *p* = 0.0089).

**Conclusions:**

Our findings support the involvement of *NFE2L2* gene variants in ARDS susceptibility and reinforce further exploration of the role of oxidant stress response as a risk factor for ARDS in critically ill patients.

## Introduction

Acute respiratory distress syndrome (ARDS) remains a major cause of death in adult intensive care units (ICUs), with most epidemiological reports mentioning a hospital mortality rate over 40 % [1]. Despite a similar pulmonary response, this complex syndrome develops as a complication of several acute disease processes, with sepsis being the most common predisposing condition [1, 2]. Damage to the alveolar–capillary membrane results in increased vascular permeability and protein-rich alveolar edema. The clinical diagnosis is made on the basis of a combination of severe hypoxemia requiring mechanical ventilation with high concentrations of oxygen, bilateral pulmonary infiltrates on chest radiographs, and reduced lung compliance [3].

Critical illness is characterized by an increased production of reactive oxygen species (ROS) [4]. Under physiological conditions, oxygen metabolism generates small amounts of ROS, although the cells have several antioxidant mechanisms against oxidative damage. A disruption of oxidant–antioxidant balance is likely to play a role in the pathogenesis of several inflammatory conditions, including sepsis and ARDS [5]. The nuclear factor erythroid 2-like 2, also known as NRF2 or *NFE2L2*, plays a central role in the antioxidant mechanisms against ROS. NFE2L2 is a member of the Cap’n’Collar basic leucine zipper transcription factor family and constitutes a hub, controlling the expression of several genes involved in regulating cellular antioxidant levels and detoxification [6]. The *NFE2L2* gene maps onto chromosome 2 at 2q31. Upon activation by an increase in cellular levels of ROS, NFE2L2 translocates to the nucleus and binds to the antioxidant response element (ARE), inducing the transcription of NFE2L2-regulated genes [6]. In previous positional cloning studies in experimental animals, researchers have identified *NFE2L2* as a candidate gene for hyperoxia-induced lung injury susceptibility [7, 8]. These results were validated in humans, in whom common single-nucleotide polymorphisms (SNPs) were identified by resequencing analysis and candidate SNP functionality was proven in cell lines. In addition, the association of common variants with ARDS susceptibility and mortality has been reported recently [9–11].

In the present study, we aimed to assess the association of common genetic variants in *NFE2L2* with ARDS in patients admitted with severe sepsis in a Spanish network of post-surgical and critical care units.

## Methods

This study is part of an ongoing research program in which the role of genetic factors on ARDS susceptibility is being analyzed. This study was approved by the external scientific committee and advisory committee of experts on ethical, economic, environmental, legal, and social affairs at the Spanish national DNA biobank (National DNA Bank Carlos III); the ethics committee at the coordinating center (Hospital Universitario Nuestra Señora de Candelaria, Tenerife, Spain); and the institutional review boards of participating hospitals (Hospital Clínico de Santiago de Compostela, Hospital General de León, Hospital Universitario Río Hortega, Fundació Althaia, Hospital Clinic de Barcelona, Hospital NS del Prado, Hospital Vírgen de la Luz, and Hospital General de Ciudad Real). Informed consent was obtained from all subjects or from their appropriate surrogates.

### Study design

We used a case–control study design with 1222 DNA samples from unrelated individuals. We enrolled 322 patients with a diagnosis of severe sepsis [12] and ARDS who were admitted into a multidisciplinary network of post-surgical and ICUs in Spain (see Appendix). All patients were mechanically ventilated. ARDS was defined according to the Berlin criteria [13]. For the purpose of this study, patients with mild, moderate, and severe ARDS were analyzed as a single group of patients with ARDS. Although the selection of controls remains a challenge [14], we preferred to use population-based subjects as controls instead of using patients at risk, because the former minimize the introduction of selection and Berkson bias [15, 16] without sacrificing genotype compliance with Hardy-Weinberg equilibrium (HWE) expectations and therefore provide an additional quality control on genotyping. The population-based control group included DNA samples from 900 unrelated adults (control/case ratio of approximately 3) provided by the Spanish national DNA biobank [17]. A health survey was obtained from all control subjects, and none of them had a history of respiratory diseases.

We recorded basic demographic data, severity of illness scores, and clinical information, including source of infection and development of organ failure until ICU discharge. Blood samples for genotyping analysis were collected within the first 24 hours of meeting the criteria for severe sepsis.

### Genotyping

Genomic DNA was extracted from whole blood using an illustra GFX PCR DNA kit (GE Healthcare Life Sciences, Little Chalfont, UK). We followed current guidelines for DNA polymorphism association studies [18]. Sample size was based on an a priori power calculation with Quanto software (http://biostats.usc.edu/Quanto.html) [19] to attain 80 % power for an allele frequency of 10 % and an effect size (odds ratio [OR]) of 1.5, assuming an ARDS incidence of 7.2 new cases per 100,000 population per year in the Spanish population [3].

We first selected a set of six tagging SNPs (tSNPs) using TagIT software [20]. This approach provided a mean coverage of *r*^2^ > 0.85 for the common gene variation [minor allele frequency (MAF) ≥5 %] based on the information on the European population derived from the 1000 Genomes Project (1KGP) [21]. Genotyping was performed using the MassARRAY iPLEX Gold™ platform (Sequenom, San Diego, CA USA) and TaqMan™ allelic discrimination assays (Applied Biosystems, Foster City, CA, USA). Individual SNP genotype calls were automatically generated using Sequenom TYPER 3.4™ software. TaqMan genotyping was used for the SNP rs6706649, and performed using a 7500 Fast Real-Time PCR System (Life Technologies, Carlsbad, CA, USA). Genotyping was done blinded to control and case status. DNA from two HapMap individuals and approximately 7 % of the samples was genotyped in duplicate to monitor genotyping quality. The estimated overall genotype concordance among duplicates was 100 % (95 % confidence interval [CI], 95.0–100 %). Twenty-nine control subjects and one patient with ARDS were excluded from downstream analyses because of a low completion rate (<80 %).

### Statistical analysis

Clinical and demographic data were analyzed with χ^2^ tests for categorical variables and the Mann–Whitney *U* test for ordinal data using R 3.01 software [22]. Quality control and deviations from HWE in genotyped SNPs were assessed using SNPing software [23]. SNP imputation using data from European individuals from 1KGP phase I (May 2011) [21] was performed using MaCH 1.0 software [24]. Association testing was conducted for allele dosages using Mach2dat [24] for those SNPs showing MAF ≥5 % and squared correlation between imputed and observed genotypes (*R*^2^) ≥0.3. The independence of SNP associations was examined with conditional regression analysis using the R statistical software package. To control for type I errors arising from multiple hypothesis testing, a false discovery rate (FDR) was calculated by means of qvalue [25]. A FDR threshold of 0.05 was established to declare significance. Pairwise *r*^2^ values were calculated using Haploview 3.32 [26] to assess the linkage disequilibrium (LD) between SNPs based on data deposited for Europeans in the 1KGP database. Evaluation of functionality of associated SNPs was performed with the online software HaploReg v3 [27] on the basis of empirical data from the ENCODE project [28] with the aim of identifying functional elements in the human genome sequence. Specifically, we focused our attention on ENCODE experiments performed on cell lines and tissues obtained from lungs, lung developmental stages, and endothelium.

## Results

### Characteristics of patients

Demographic and clinical data from the 321 ARDS patients and 871 population-based control subjects with SNP completion rate ≥80 % are summarized in Table 1. The overall mortality rate at discharge from the ICU was 36.3 %. In 35 % of patients, no pathogens were identified as the causative microorganism for sepsis, although all of them had an identified or highly suspected site of infection, a finding that is in accordance with published data [29]. The most common sites of infection were the lung, the abdominal cavity, and the gastrointestinal tract.

**Table 1:** Demographic and clinical characteristics of the study sample

Characteristic		ARDS patients	Controls	*p* value
		(n = 321)	(n = 871)	
Sex (% male)		63.2	59.6	0.242^a^
Median age, yr (P_25_–P_75_)		67 (55–75)	41 (32–49)	<0.001^b^
Hypertension (%)		42.4	3.1	<0.001^a^
Smoker (%)		27.9	31.2	0.422^a^
Previous surgery (%)		65	NA	
Ischemic cardiac disease (%)		9.0	NA	
Source of sepsis (%)				
	Pulmonary	41.9	NA	
	Extrapulmonary	58.1	NA	
Pathogen (%)				
	Gram-negative	29.3	NA	
	Gram-positive	21.1	NA	
	Mixed	5.7	NA	
	Polymicrobial	3.3	NA	
	Virus	2.8	NA	
	Fungi	2.8	NA	
	Negative blood cultures	35.0	NA	
Organ dysfunction (%)				
	Circulatory	58.0	NA	
	Renal	43.6	NA	
	Hepatic	21.4	NA	
	Neurologic	20.8	NA	
	Coagulation	18.9	NA	
APACHE II, mean (P_25_–P_75_)		22 (17–27)	NA	
PaO_2_/FiO_2_ mean,^c^ mmHg (P_25_–P_75_)		206 (124–255)		
ICU mortality (%)		36.3	NA	

*APACHE II* Acute Physiology and Chronic Health Evaluation II, *ICU* intensive care unit, *NA* not applicable, *P*_*25*_ percentile 25, *P*_*75*_ percentile 75, *PaO*_*2*_ */FiO*_*2*_ ratio of partial pressure arterial oxygen and fraction of inspired oxygen

^a^χ^2^ test

^b^Mann–Whitney *U* test

^c^At the time of onset

### Genotype frequencies and association with acute respiratory distress syndrome

**Fig. 1:**
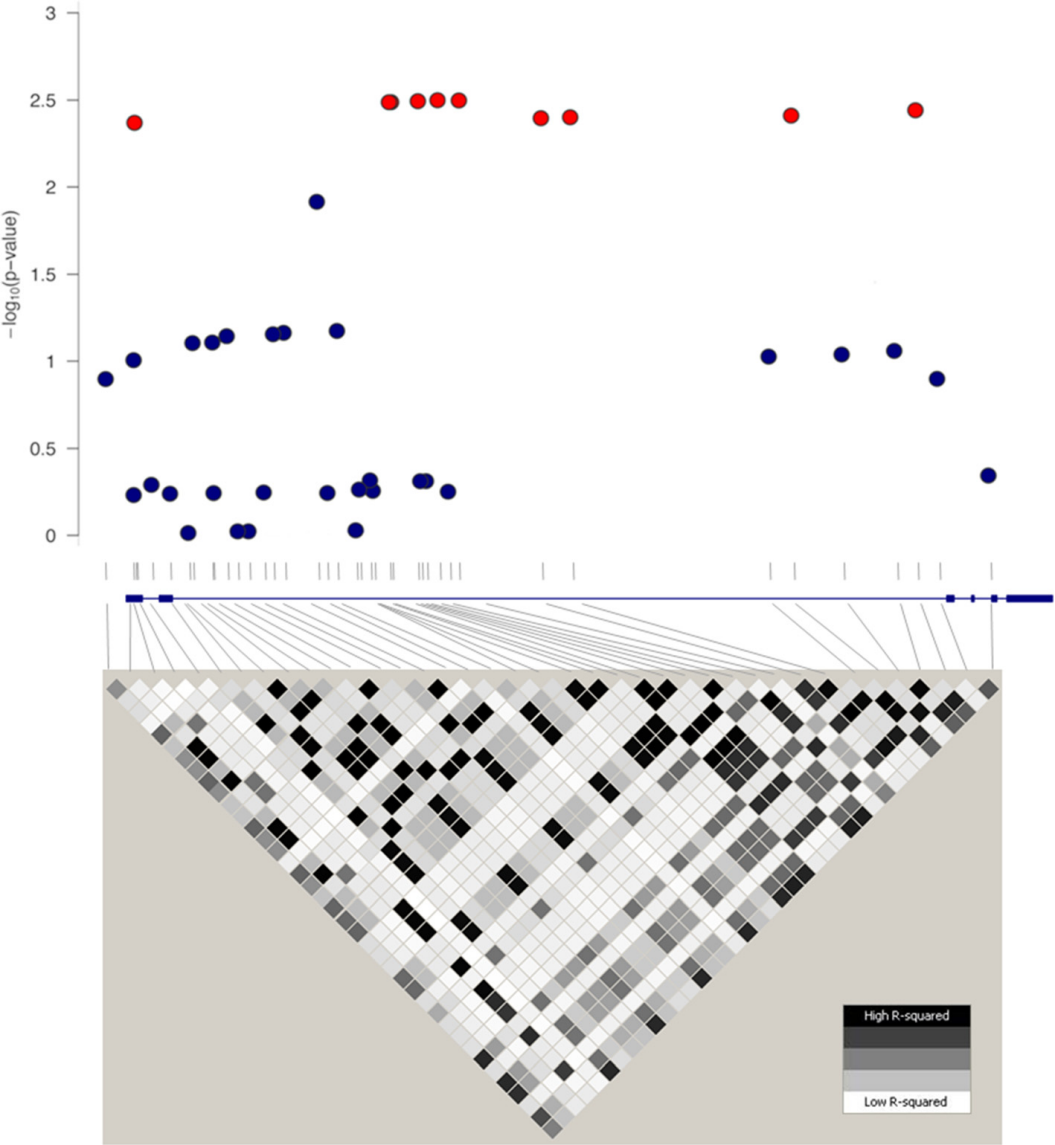
Regional plot of association results. *Upper panel:* The *y*-axis represents the − log_10_-transformed *p* values for association tests. The *x*-axis represents the approximate location of the 40 single-nucleotide polymorphisms (SNPs) tested for association relative to the gene. *Red circles* depict associated SNPs after multiple comparison adjustments. *Lower panel:* This linkage disequilibrium (LD) plot is based on pairwise *r* ^2^ values among SNPs from the European population of the 1000 Genomes Project. Each diamond of the LD plot represents an *r* ^2^ value between two SNPs, schematically symbolized by a color gradient ranging from *black* (*r* ^2^ = 1, corresponding to complete LD) to *gray* (1 < *r* ^2^ < 0, moderate LD) and *white* (*r* ^2^ = 0, absence of LD)

All the tSNPs had a genotype completion rate >95 %. In the control group, none of the six tSNPs deviated significantly from HWE expectations (Table 2). After imputation, association testing was conducted for 40 SNPs with MAF ≥5 % and *R*^2^ ≥ 0.3. Finally, a total of 10 SNPs, all non-coding, were significantly associated with ARDS susceptibility after multiple testing adjustments (FDR = 0.036), with the top hit being rs4243387 (OR for C allele = 1.93; 95 % CI, 1.19–3.12; *p* = 0.0068) (Table 3). Although the 10 SNPs associated are distributed along approximately 10 kb of the gene, and though several of them relate to histone marks according to empirical data (Table 3), they constitute a single association signal, owing to the strong LD among them (0.75 ≤ *r*^2^ ≤ 1) (Fig. 1). In fact, association analyses using regression models accounting for the top hit rendered the remaining SNPs non-significant (Table 4). In addition, of the 10 associated SNPs that were not independent from each other, it is worth noting that the rs6721961 was among the associated SNPs showing an OR of 1.93 per each addition of a T allele (95 % CI, 1.17–3.18; *p* = 0.0089). This SNP is located at −178 bp from the transcription start site of the gene [30] and was previously associated with trauma-induced ARDS, although it was described at position −617 bp in that publication [9].

**Table 2:** Location, allele frequency, and quality control information for tagging single-nucleotide polymorphisms genotyped for *NFE2L2* gene

tSNPs	Alleles	Position^a^	CR (%)	MAF cases	MAF controls	HWE controls
rs35652124	T/C	177,265,344	98.3	0.250	0.274	0.231
rs6706649	C/T	177,265,342	95.2	0.115	0.126	0.341
rs2364722	A/G	177,260,058	99.6	0.254	0.280	0.152
rs72946143	T/C	177,253,423	100	0.044	0.067	0.270
rs1806649	C/T	177,253,423	99.3	0.300	0.317	0.481
rs6726395	A/G	177,238,500	99.1	0.469	0.494	0.378

*CR* completion rate, *HWE* Hardy-Weinberg equilibrium *p* value, *MAF* minor allele frequency

^a^According to National Center for Biotechnology Genome Reference Consortium NCBI build GRCh38

**Table 3:** Summary of *NFE2L2* variants associated with acute respiratory distress syndrome susceptibility with false discovery rate <0.05

Position^a^	SNPs	Functionality^b^	Minor allele	MAF	*R* ^2^	OR (95 % CI)	*p* value
177,265,308	rs6721961	5′ flanking	T	0.111	0.32	1.93 (1.17–3.18)	0.0089
		Histone mark, DHS					
177,255,662	rs10188193	Intron 1	T	0.110	0.33	1.95 (1.19–3.17)	0.0071
177,255,583	rs10188107	Intron 1	T	0.110	0.33	1.95 (1.19–3.17)	0.0071
177,254,567	rs10497511	Intron 1	G	0.110	0.33	1.95 (1.19–3.17)	0.0070
		DHS					
177,253,821	rs2001297	Intron 1	C	0.110	0.33	1.95 (1.20–3.16)	0.0069
		Histone mark					
177,253,036	rs4243387^c^	Intron 1	C	0.112	0.34	1.93 (1.19–3.12)	0.0068
		Histone mark					
177,249,903	rs10930781^c^	Intron 1	A	0.106	0.35	1.90 (1.17–3.12)	0.0085
177,248,755	rs1962142^c^	Intron 1	A	0.103	0.33	1.96 (1.18–3.23)	0.0083
		Histone mark					
177,240,415	rs2364720	Intron 1	A	0.106	0.35	1.90 (1.17–3.09)	0.0082
		Histone mark					
177,235,696	rs2001350^c^	Intron 1	C	0.103	0.32	2.00 (1.20–3.35)	0.0075
		Histone mark					

*CI* confidence interval, *DHS* DNase I hypersensitivity site, *FDR* false discovery rate, *MAF* minor allele frequency, *OR* odds ratio, *R* ^2^ squared correlation between imputed and observed genotypes, *SNP* single-nucleotide polymorphism

^a^According to National Center for Biotechnology Genome Reference Consortium NCBI build GRCh38

^b^Functionality obtained from HaploReg v3 [27]

^c^SNPs associated with primary graft dysfunction in Cantu et al. [11]

**Table 4:** Conditional regression results accounting for the effect of rs4243387

SNP	Univariate association *p* value	Conditional regression *p* value
rs10188107	0.0071	0.464
rs10188193	0.0071	0.498
rs10497511	0.0070	0.580
rs10930781	0.0085	0.929
rs1962142	0.0083	0.956
rs2001297	0.0069	0.678
rs2001350	0.0075	0.940
rs2364720	0.0082	0.980
rs6721961	0.0089	0.292

*SNP* single-nucleotide polymorphism

## Discussion

This study is the first examining, the association of common variants of *NFE2L2* gene with susceptibility to ARDS among patients with severe sepsis, finding an association of 10 SNPs with this syndrome. Although these SNPs were widely distributed across the gene, all of them showed strong LD with each other. One of the associated SNPs, rs6721961, which showed a minor allele frequency of 11.1 % in this sample, was located in the promoter region of the gene, and its T-allele had been previously linked to a reduction in functionality that limited the NFE2L2 triggering of the antioxidative response [9]. Consistent with those findings, we found that the T allele at rs6721961 conferred greater risk for ARDS susceptibility in patients with sepsis than in healthy subjects. A functional evaluation of the associated SNPs with empirical data from the ENCODE project revealed that seven of them are located in histone marks and/or on DNase I hypersensitivity sites (Table 3). Specifically, the SNP rs6721961 locates in a promoter histone mark in lung fibroblasts and fetal lung. It is also located in a DNase I hypersensitivity site, as reported in an epithelial cell line derived from a lung carcinoma tissue. Given that chromatin modifications on histone marks are critically involved in the regulation of gene expression and that these regions tend to collocate with DNase-sensitive sites in transcriptional start sites [31], this evidence highlights the key role of rs6721961 in the regulation of *NFE2L2* expression. Overall, our results highlight the importance of *NFE2L2* gene variants in modulating the response to oxidative damage among critically ill patients.

Reduction–oxidation (redox) balance is particularly important in the airways because they represent the first contact with environmental oxidants. Generation of ROS has been implicated in the pathogenesis of many acute and chronic pulmonary diseases, including ARDS [32], and it is a common condition among critically ill patients that results in the development of multiple organ system failure [33]. In this respect, researchers in several studies have reported the presence of oxidative damage in patients with sepsis [34, 35]. High levels of protein oxidation have been found, both in plasma and in bronchoalveolar fluids, in early stages of severe sepsis development and during major trauma [36]. NFE2L2 constitutes a hub and a master regulator of detoxifying systems, such as catalase, superoxide dismutase, and glutathione peroxidase, that are critically involved in protecting the cells against oxidative stress [6]. Using experimental animal models where this transcription factor was disrupted allowed the identification of dependent genes that are critical in pulmonary protection and confirmed that its disruption promotes susceptibility to several prooxidant-induced lung diseases, primarily owing to decreased levels of the basal and inducible expression of several antioxidant enzymes [37–40].

The importance of redox balance in the pathogenesis of ARDS, as well as the implication of *NFE2L2* in disease susceptibility or outcome, is supported by the identification of variants in a few genes involved in the oxidative stress response previously associated with ARDS susceptibility or outcome [41, 42]. Marzec et al. [9] assessed the functionality of the SNP rs6721961 (referred to by those authors at position −617 bp) on the promoter region by means of a reporter gene assay and an electrophoretic mobility shift assay (EMSA). In the reporter gene assay, the luciferase activity for the T allele was less than half of the activity for the G allele, indicating a significant reduction of *NFE2L2* gene expression. Congruently, results derived from the EMSA showed that the formation of a protein–DNA complex was significantly diminished in the presence of the T allele, suggesting a less efficient binding of the NFE2L2 transcription factor to the ARE-like sequences of its target genes. In the same study, and consistent with these results, the presence of the T allele at rs6721961 was associated with risk for ARDS susceptibility in a nested case–control association study conducted with 30 patients with trauma-induced ARDS and 60 matched at-risk control subjects. A fixed-effects meta-analysis combining these results with those derived from our study confirmed the concordance of effects at the SNP level, showing an OR for the T allele of 2.18 (95 % CI, 1.35–3.50; *p* = 0.0013). The same SNP was recently found to be associated with 28-day mortality in a nested case–control study that included 224 patients with ARDS from a cohort of 750 patients with systemic inflammatory response syndrome [10]. Also, Cantu et al. [11] found a few *NFE2L2* SNPs associated with primary graft dysfunction, a specific form of ARDS developed within 72 hours after lung transplantation. Four of those SNPs (rs10930781, rs1962142, rs2001350, and rs4243387) were also significantly associated with ARDS in our study.

The reliability of association findings can be assessed only by replicating the results in independent samples. Such an effort has been widely recognized as a major gap in the field [14, 43]. In this respect, the present study can be considered a SNP-level replication of previous findings in a large series of patients, although the precipitating injury was severe sepsis instead of trauma. As a result of this, although ancestry adjustments were not implemented, a confounder effect due to the presence of population stratification in this study would be minimal. Besides, we acknowledge some minor limitations. First, although the study sample provided 80 % power to detect a minimum risk of 1.5, we recognize that this sample size is limited to detect smaller effect sizes that are expected on average for complex traits [44]. Second, we assessed only the common variation within the *NFE2L2* gene, and they would contribute to an explanation of only a modest fraction of the genetic component of the syndrome. Third, the use of population-based controls instead of at-risk controls precludes deducing whether the *NFE2L2* gene is associated with ARDS or with the underlying condition (e.g., severe sepsis). However, whatever the case, the fact that our results replicated previous findings from a study using at-risk controls strongly supports that this gene is directly involved in ARDS susceptibility.

## Conclusions

We provide evidence implicating common *NFE2L2* gene variants in ARDS susceptibility, reinforcing further explorations of the role of oxidant stress response as a risk factor for ARDS in critically ill patients. Research in this field will eventually translate into potentially useful information by identifying new pathways and novel therapeutic approaches, and also by developing predisposition biomarkers to stratify at-risk patients, thus facilitating personalized patient assessment and better patient management.

## Key messages

- A number of common variants of the *NFE2L2* gene are associated with ARDS in patients with severe sepsis.
- One of the associated SNPs is located in the promoter region and has been proven to modify the promoter activity of the *NFE2L2* gene.
- Our study supports the role of the oxidant stress response as a risk factor for ARDS in critically ill patients, irrespective of the precipitating injury.

## Appendix

### GEN-SEP investigators

Jesús Villar, Rosa L. Fernández (Hospital Universitario Dr. Negrín, Las Palmas de Gran Canaria); Carlos Flores, Maria Pino-Yanes, Marialbert Acosta-Herrera, Lina Pérez-Méndez, Almudena Corrales, Elena Espinosa, David Domínguez (Hospital Universitario Nuestra Señora de Candelaria, Santa Cruz de Tenerife); Alfonso Ambrós, Rafael del Campo (Hospital General de Ciudad Real, Ciudad Real); Rafael Fernández, Carles Subirá, José A. Rodríguez (Fundació Althaia, Barcelona); Aurora Baluja, Julián Álvarez (Hospital Clínico Universtario de Santiago de Compostela, La Coruña); José M. Añón, Elena González, Oscar Hernández, Rosario Solano, Javier Pérez-Crespo, Paola Arellano (Hospital Virgen de La Luz, Cuenca); Ramón Adalia, Eli Zavala, Julia Martínez, Antoni Torres, Joan Badia (Hospital Clínic, Barcelona); and Francisco Alba, Ruth Corpas (Hospital Nuestra Señora del Prado, Toledo).

### GRECIA investigators

Jesús Blanco, Arturo Muriel (Hospital Universitario Río Hortega, Valladolid); Victor Sagredo, Juan C. Ballesteros (Hospital Clínico Universitario de Salamanca, Salamanca); Francisco Taboada, Guillermo M. Albaiceta (Hospital Central de Asturias, Oviedo); Francisco Gandía, Felipe Bobillo (Hospital Clínico Universitario de Valladolid, Valladolid); Luis Tamayo (Hospital Río Carrión, Palencia); A.G. Labattut (Hospital General de Soria, Soria); Demetrio Carriedo, Javier Collado, Francisco J. Diaz (Hospital General de León, León); M. Valledor, Maite Antuña (Hospital San Agustín, Avilés); M. de Frutos (Hospital General Yagüe, Burgos); María J. López, José J. Cortina (Hospital General de Segovia, Segovia); Teresa Saldaña, Ana Caballero, Teresa Álvarez (Hospital Virgen de la Concha, Zamora); and Braulio Álvarez, José Sandoval (Hospital del Bierzo, Ponferrada).

## Abbreviations

1KGP: The 1000 genomes project
APACHE II: Acute Physiology and Chronic Health Evaluation II
ARDS: Acute respiratory distress syndrome
ARE: Antioxidant response element
CI: Confidence interval
CR: Completion rate
DHS: DNase I hypersensitivity site
EMSA: Electrophoretic mobility shift assay
FDR: False discovery rate
HWE: Hardy-Weinberg equilibrium
ICU: Intensive care unit
LD: Linkage disequilibrium
MAF: Minor allele frequency
*NFE2L2*: Nuclear factor erythroid 2-like 2
OR: Odds ratio
PaO_2_/FiO_2_: Ratio of partial pressure arterial oxygen and fraction of inspired oxygen
Redox: Reduction–oxidation
ROS: Reactive oxygen species
SNP: Single nucleotide polymorphism
tSNP: Tagging single-nucleotide polymorphism
